# Research Review: Internalising symptoms in developmental coordination disorder: a systematic review and meta‐analysis

**DOI:** 10.1111/jcpp.13001

**Published:** 2018-11-28

**Authors:** Serif Omer, Ana M. Jijon, Hayley C. Leonard

**Affiliations:** ^1^ School of Psychology University of Surrey Guildford Surrey UK

**Keywords:** Developmental coordination disorder, internalising symptoms, depression, anxiety, mental health, meta‐analysis

## Abstract

**Background:**

Developmental coordination disorder (DCD) affects 5%–6% of children. There is growing evidence that DCD is associated with greater levels of internalising symptoms (i.e. depression and anxiety). This is the first systematic review and meta‐analysis to explore the magnitude of this effect, the quality of the evidence and potential moderators.

**Methods:**

A systematic search was conducted to identify studies reporting a comparison between individuals with DCD/probable DCD and typically developing (TD) individuals on measures of internalising symptoms. A pooled effect size (Hedges *g*) was calculated using random‐effects meta‐analysis. Study quality, publication bias and potential moderators of the effect were explored.

**Results:**

Twenty studies, including a total of 23 subsamples, met the inclusion criteria, of which 22 subsamples were included in the meta‐analysis (DCD:* n* = 1123; TD:* n* = 7346). A significant, moderate effect of DCD on internalising symptoms was found (*g *=* *0.61). This effect remained robust after accounting for publication bias and excluding lower quality studies. The effect was significantly larger in studies utilising a cross‐sectional design (vs. longitudinal), convenience sampling (vs. population screening) and a majority male sample.

**Conclusions:**

The findings demonstrate that individuals with DCD experience greater levels of internalising symptoms than their peers. This highlights the importance of routine screening for emotional difficulties in DCD, raising awareness of the condition in mental health services and developing psychosocial interventions that extend beyond a focus on motor impairments. However, there is a need for higher quality, longitudinal studies to better understand the causal relationship between DCD and internalising symptoms.

## Introduction

Developmental coordination disorder (DCD) is a neurodevelopmental disorder affecting between 5%–6% of children and is characterised by significant impairment to an individual's ability to perform everyday motor tasks (American Psychiatric Association [APA], 2013). This can include difficulties with self‐care (e.g. tying shoelaces), academic tasks (e.g. handwriting) and leisure activities (e.g. catching a ball). A diagnosis of DCD is based on four criteria (APA, 2013): (a) performance in motor coordination tasks is substantially below expectation given the person's age and opportunities; (b) the motor coordination difficulties significantly interfere with activities of daily living or academic achievement; (c) difficulties began in the early developmental period; and (d) the difficulties cannot be attributed to an intellectual disability or neurological condition (e.g. cerebral palsy).

Despite its prevalence, DCD often goes unrecognised and is poorly understood among healthcare and education professionals (Gaines, Missiuna, Egan, & McLean, 2008; Wilson, Neil, Kamps, & Babcock, 2013). This is of concern given that DCD has been found to have a significant impact not just on an individual's motor abilities, but across a wide range of psychological, cognitive, physical and social domains (Zwicker, Harris, & Klassen, 2013). There is also evidence that the impact of DCD across these domains persists into adulthood (Cousins & Smyth, 2003; Hill, Brown, & Sorgardt, 2011).

One area that has received increasing attention is the impact of DCD on mental health, specifically internalising symptoms (i.e. depression and anxiety). There is growing evidence that individuals with DCD have elevated levels of internalising symptoms compared to their typically developing (TD) peers (Mancini, Rigoli, Cairney, Roberts, & Piek, 2016; Mancini, Rigoli, Roberts, & Piek, 2018). Research has also found associations between motor ability and internalising symptoms in community samples of TD children and adults (Poole et al., 2016; Rigoli, Piek, & Kane, 2012; Wilson, Piek, & Kane, 2013), an increased risk of psychiatric disorders in individuals with DCD (Rasmussen & Gillberg, 2000), and impaired motor ability in individuals with common psychiatric disorders (Damme, Simons, Sabbe, & van West, 2015).

Understanding the link between DCD and mental health has important implications for assessment and intervention with this population, including at schools, physical health services and mental health services. To date, several reviews have summarised the findings on internalising symptoms in DCD (Caçola, 2016; Mancini et al., 2016, 2018; Missiuna & Campbell, 2014). However, they consist of narrative summaries only. There are also inconsistent findings, with some studies finding no significant effect (Davis, Ford, Anderson, & Doyle, 2007; King‐Dowling, Missiuna, Rodriguez, Greenway, & Cairney, 2015) and others a large effect (Dewey, Kaplan, Crawford, & Wilson, 2002; Pratt & Hill, 2011). A systematic search, synthesis and critical appraisal of the evidence can provide a more rigorous understanding of this relationship (Mancini et al., 2016, 2018). A meta‐analysis to pool the findings would provide a more accurate understanding of whether individuals with DCD do indeed experience greater internalising symptoms than their peers and would provide insight into the magnitude of this difference.

Studies also vary greatly in their design, participants, measures and methodological quality. Whereas some studies have recruited participants with a confirmed diagnosis of DCD, others have included only those identified as ‘probable DCD’ (i.e. based on parent‐report or performance‐based screening measures without comprehensive assessment of all diagnostic criteria). Studies also differ in how well they controlled for confounding variables, whether they employed a longitudinal or cross‐sectional design and whether they recruited participants through population‐based screening or convenience sampling. These differing methodological factors could all impact on the quality of a study and, thus, the magnitude of the effect identified (Sanderson, Tatt, & Higgins, 2007). There is some evidence to suggest the impact may be greater in adolescents compared to younger children (Piek et al., 2007; Skinner & Piek, 2001), in males (Sigurdsson et al., 2002) and in individuals with comorbid attention‐deficit hyperactivity disorder (ADHD; Missiuna et al., 2014; Piek et al., 2007), although these factors are unlikely to explain all the variance in the relationship between DCD and internalising symptoms. Research has also highlighted differences between parent‐ and child‐reported measures of internalising symptoms, with parents often under‐reporting difficulties (Cantwell, Lewinsohn, Rohde, & Seeley, 1997). A meta‐analytic approach allows for an investigation into the potential sources of heterogeneity across studies, which could help to guide future research and intervention.

The aim of this paper, therefore, was to answer the questions: do individuals with DCD experience significantly greater levels of internalising symptoms than TD individuals, and what is the magnitude of this difference? The specific objectives were to conduct a systematic review and meta‐analysis of studies that compared individuals with DCD to TD individuals on measures of internalising symptoms; to appraise the quality of the evidence; and to explore which factors moderate the effect. The focus was on severity levels of internalising symptoms, as opposed to rates of actual diagnosis, given that most studies have adopted severity outcome measures, and given that data on diagnostic rates may obscure important differences in actual symptoms.

## Method

The review was protocol‐driven and carried out in accordance with recommended guidelines for systematic reviews (Moher, Liberati, Tetzlaff, Altman, & The Prisma Group, 2009) and meta‐analyses of observational studies (Stroup et al., 2000).

### Eligibility criteria

In line with recommended guidelines, broad inclusion criteria were used with the aim to later explore the impact of specific design features. Articles were eligible if they: (a) included participants, of any age, with a confirmed diagnosis of DCD according to DSM‐IV or DSM‐5 criteria; or who were identified as having motor coordination difficulties consistent with DCD (i.e. ‘probable DCD’); (b) included a comparison group of TD individuals, as defined by the absence of diagnosed or suspected developmental disorders at the time of the study; (c) measured levels of internalising symptoms (i.e. depression and/or anxiety) for each group using self‐report, parent‐report, teacher‐report, direct observation or clinical interview; (d) reported statistics that could be transformed into a standardised mean difference; and (e) were available in full text in English. Studies involving participants with a comorbid diagnosis (e.g. ADHD) were eligible if the motor coordination difficulties were clearly described and used as the basis for group comparison. Studies were excluded if participants’ motor difficulties were attributed to another developmental difficulty or medical diagnosis (e.g. cerebral palsy). For studies using the label ‘dyspraxia’, they were required to refer to overall motor impairment and not just oromotor difficulties and gesture. Studies were also excluded if the outcome only included rates of psychiatric diagnoses (i.e. they did not report on a measure of the severity of symptoms).

If separate studies included overlapping samples, priority was given to the study with the best control of important confounders (i.e. age and gender) or the study that allowed for the most detailed exploration of moderating factors (e.g. outcomes reported separately by gender or age group). Where the same participants were included but different subtests reported, data were combined (Borenstein, Hedges, Higgins, & Rothstein, 2009).

### Search strategy

Studies were identified through a systematic search of Medline, PsychInfo, CINAHL, ERIC and Web of Science. Unpublished studies were searched using ProQuest Dissertations and Theses and Open Grey. The latest search was completed on 3rd March 2018. The search included terms related to DCD combined with terms related to internalising symptoms (see Appendix S1). Titles and abstracts were screened by one reviewer (SO), with 25% cross‐checked by a second (AJ; per cent agreement = 97.5%, Cohen's kappa = .65). The full texts of all potentially relevant articles were then screened independently by two reviewers (SO & AJ), with disagreement resolved by consensus and discussion with a third (HL; per cent agreement = 94.5%, kappa = .87). The bibliographies of the included studies and relevant review articles were screened and their citations were tracked to identify additional studies. The first authors of the included articles were contacted to identify any further eligible studies or to clarify missing information.

### Data extraction

Two researchers (SO & AJ) performed data extraction for all included studies and inconsistencies were discussed until consensus was reached. Interrater reliability was good for both categorical (per cent agreement = 96%–100%; kappa = .89–1.00) and continuous (intraclass correlation coefficient = 1.00) data.

The following information was extracted: author, publication year, country, design (cross‐sectional; longitudinal), population, sampling procedure (population‐based screening; selective/convenience sample), criteria for DCD (confirmed DCD; probable DCD), criteria for TD, number of participants, gender (percentage male), age (mean and range), comorbid ADHD diagnosis (ADHD assessed and excluded from the sample; ADHD assessed and included; ADHD not assessed), measures of internalising symptoms, internalising construct (depression; anxiety; overall internalising), reporter (self‐report; parent‐report; teacher‐report; clinician/researcher‐report) and scores (means and standard deviations, other relevant statistics).

If multiple informants or measures were used to assess internalising symptoms, they were extracted separately so that they could be pooled. Preference was given to data adjusted for important confounders (e.g. gender, age) if not matched by design. However, where studies also adjusted for additional variables (e.g. intelligence), the unadjusted scores were preferred to ensure comparability across studies (Voils, Crandell, Chang, Leeman, & Sandelowski, 2011). Where findings were reported separately for subgroups (e.g. gender, age groups), these data were extracted separately as subsamples. Where separate groups were included for confirmed and probable DCD, only the confirmed DCD group was extracted. Where separate groups were included for comorbid DCD/ADHD and DCD‐only, the DCD‐only group was extracted.

### Study quality

An adapted version of the Newcastle–Ottawa Scale (NOS) was used to assess study quality (Wells et al., 2011). Two reviewers (SO & AJ) conducted ratings independently, with disagreement discussed until consensus was reached (per cent agreement = 91%–100%; kappa = .82–1.00). Each study was rated on representativeness of the DCD group (i.e. population screening), selection of the control group (i.e. same population as DCD), ascertainment of DCD diagnosis (i.e. confirmed DCD); control for baseline internalising (for longitudinal studies), comparability of groups (i.e. control for confounders), measurement of internalising symptoms (i.e. validated measures), length of follow‐up (for longitudinal studies) and completeness of follow‐up (for longitudinal studies only).

For the DCD criteria to be rated as ‘Confirmed DCD’, the study must have assessed motor skills as being below the 15th percentile using performance‐based measures (Criterion A; Blank, Smits‐Engelsman, Polatajko, & Wilson, 2012), as having a significant impact on activities of daily living or academic achievement (e.g. questionnaires or interview; Criterion B), and ruled out intellectual disability and other neurological conditions (e.g. by interview, performance measures, medical reports; Criterion D). Alternatively, they could have cross‐checked medical records for diagnosis. Given that many studies were published prior to publication of the DSM‐5 and the introduction of Criterion C, it was not essential that studies established whether participants met this criterion (i.e. if difficulties began in the early developmental period). The confounding factors of age and gender were considered to be the most important for comparability of study groups (Twenge & Nolen‐Hoeksema, 2002). The NOS satisfies relevant guidelines (Sanderson et al., 2007) and is recommended for systematic reviews of observational studies (Deeks et al., 2003).

### Statistical analysis

The main analysis was performed using Review Manager 5.3 software (The Nordic Cochrane Centre, The Cochrane Collaboration, Copenhagen).

#### Summary effect

The standardised mean difference (SMD; Hedges *g*) and its 95% confidence interval were calculated for each study (or subsample) separately. SMD's around 0.2 can be considered as small, 0.5 as moderate and 0.8 as large. Where studies reported on multiple measures of internalising, a pooled effect size and variance were calculated (Borenstein et al., 2009). Effect sizes were weighted according to the inverse of their variance to ensure that more precise estimates influence overall effect size most heavily and to reduce the effect of the upwardly biased estimates of smaller studies (Hedges & Olkin, 1985). Random‐effects meta‐analysis was used to calculate a summary effect for total internalising symptoms across all studies and its 95% confidence interval.

#### Heterogeneity

*Q*‐statistics were used to assess for heterogeneity and the *I* ^2^ statistic to quantify the proportion of the variance due to heterogeneity. Moderators were explored to identify potential sources of heterogeneity. The following moderators were explored: design (longitudinal vs. cross‐sectional), gender (>50% male vs. ≤50% male in DCD group), age (included adolescents ≥12 vs. no adolescents), comorbid ADHD (assessed and excluded vs. not assessed, or assessed but included), sampling strategy (population screen vs. selective/convenience); selection of DCD group (confirmed vs. probable DCD), controlled for confounders (age and gender controlled vs. uncontrolled), reporter (self‐report vs. informant‐report) and type of internalising (overall internalising vs. depression vs. anxiety). The significance of moderators was tested using *Q*‐statistics.

#### Publication bias

Publication bias was assessed visually using a funnel plot. Egger's test was used to statistically check for publication bias. Duval and Tweedie's trim and fill procedure was used to compute an adjusted effect size by imputing the effect of smaller, unpublished studies (Duval & Tweedie, 2000). Finally, Rosenthal's (1979) Fail‐safe *N* was calculated to determine the number of studies with an average effect size of 0 that would have to be included to produce a nonsignificant result. This number should exceed 5*k* + 10 (where *k* is the number of studies).

#### Sensitivity analysis

A *s*ensitivity analysis was conducted to calculate a pooled effect size excluding lower quality studies (i.e. those not meeting at least five criteria on the NOS).

## Results

### Search results

**Figure 1:**
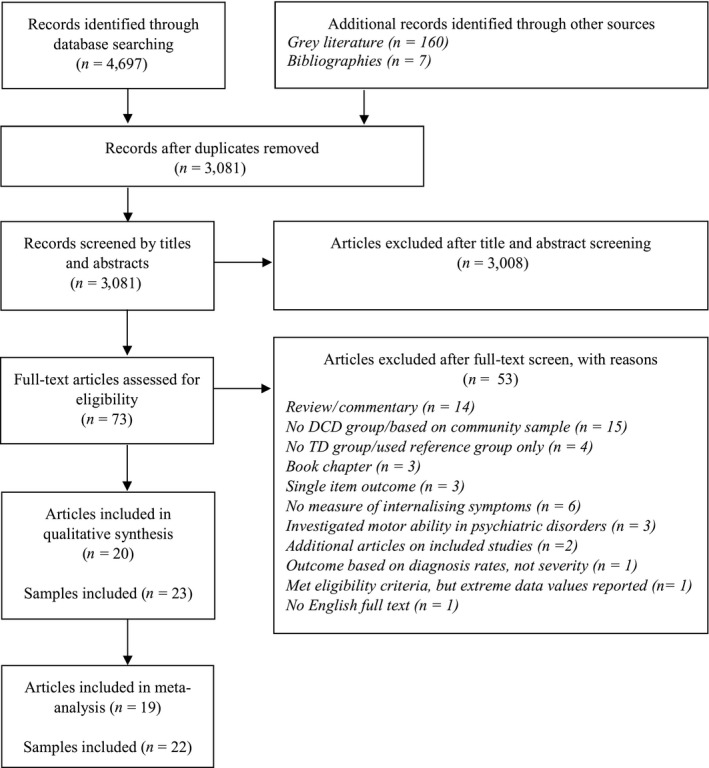
PRISMA flow diagram summarising the search process

The search identified 20 studies meeting the inclusion criteria, consisting of 23 eligible subsamples (two studies reported outcomes separately for males and females, one study for children and adolescents; hereafter treated as separate studies). The search process is summarised in Figure 1.

It should be noted that two articles reported outcomes at multiple time points for the same longitudinal study (Harrowell, Hollén, Lingam, & Emond, 2017; Lingam et al., 2012). The data from the latter time point were used (Harrowell et al., 2017) because separate outcomes were reported for males and females, allowing for better exploration of moderators. Two articles reported data on the same cross‐sectional study (Pearsall‐Jones, Piek, Rigoli, Martin, & Levy, 2011; Piek et al., 2007), so the larger sample was included (Piek et al., 2007). One eligible study reported unusually small standard deviations for the outcome, raising concerns around its accuracy and was subsequently excluded (Tseng, Howe, Chuang, & Hsieh, 2007).

### Characteristics of the included studies

The characteristics of the included studies are summarised in Table 1. A total of 8,469 participants were included (1,123 DCD, 7,346 TD). The studies were published between 1994 and 2018. Most were from developed countries, with one study from Taiwan. Three prospective cohort studies were identified that screened for DCD in a cohort and assessed their internalising symptoms at a later follow up (Harrowell et al., 2017; male & female samples; Wagner, Jekauc, Worth, & Woll, 2016). The remaining 20 studies adopted a cross‐sectional design.

**Table 1:** Summary of characteristics of the included studies

Study	Year	Population	DCD assessment	DCD sample, *n*	TD sample, *n*	DCD age, mean (range)	TD age, mean (range)	DCD gender, % male	TD gender, % male	Excluded ADHD? (measure)	Confounders controlled	Internalising measures, (type)
Longitudinal studies												
Harrowell et al. (male sample)	2017	Population screening: Birth cohort in south‐west England, motor skills assessed age 7.	Confirmed DCD (researcher assessment)	SDQ: 109; SMFQ: NR (total sample for SMFQ: 1,374)	SDQ: 1,780; SMFQ: NR (total sample for SMFQ: 1,374)	NR (SMFQ collected at 17.5 years; SDQ at 16.5 years)	NR (SMFQ collected at 17.5 years; SDQ at 16.5 years)	100	100	No (measured, but not excluded)	Age, gender.^a^	SMFQ (self‐rated depression); SDQ – emotional subscale (self‐rated internalising)
Harrowell et al. (female sample)	2017	Population screening: Birth cohort in south‐west England, motor skills assessed age 7.	Confirmed DCD (researcher assessment)	SDQ: 59 SMFQ: NR (total sample for SMFQ: 1,803)	SDQ: 1,970 SMFQ: NR (total sample for SMFQ: 1,803)	NR SMFQ collected at 17.5 years; SDQ at 16.5 years	NR SMFQ collected at 17.5 years; SDQ at 16.5 years	0	0	No (measured, but not excluded)	Age, gender.^a^	SMFQ (self‐rated depression); SDQ – emotional subscale (self‐rated internalising)
Wagner et al.	2016	Population screening: Cohort of 6–10 year olds across Germany. Motor skills assessed age 6–10.	Probable DCD (performance‐based test)	114	823	14.35 (12–16)	14.38 (12–16)	47.8	49.3	No (assessed, not excluded)	Gender, age, baseline internalising symptoms	SDQ (parent‐rated internalising)
Cross‐sectional Studies												
Campbell et al. (male sample)	2012	Population screening: Fifth‐grade students from previous longitudinal study in Ontario, Canada.	Probable DCD (screening questionnaire)	77	77	10.9 (10–11)^b^	10.9 (10–11)^b^	100	100	No (not assessed)	Age, gender	BASC‐2 – depression subscale (self‐rated depression)
Campbell et al. (female sample)	2012	Population screening: Fifth‐grade students from previous longitudinal study in Ontario, Canada.	Probable DCD (screening questionnaire)	82	82	10.9 (10–11)^b^	10.9 (10–11)^b^	0	0	No (not assessed)	Age, gender	BASC‐2 – depression subscale (self‐rated depression)
Chen et al.	2009	Population screening: 1st–3rd grade children in greater Taipei area, Taiwan.	Confirmed DCD (researcher assessment)^c^	61	209	7.93 (NR) (range for total sample: 6.25–10.08	7.68 (NR) (range for total sample: 6.25–10.08	59.0	59.8	No (measured, not excluded)	None	CBCL – Chinese version ‐withdrawn, somatic complaints, depressed/anxious subscales (parent‐rated internalising)
Crane et al.	2017	Convenience sample: children with DCD via primary schools and charity; TD via primary schools in South London, UK.	Confirmed DCD (medical report and researcher assessment)	30	35	8.61 (7–10)	9.12 (7–10)	70.0	74.3	Yes (self‐/parent‐report)	None	SDQ‐Teacher – Emotional subscale (teacher‐rated internalising)
Davis et al.	2007	Screening of clinical sample: Cohort of children born with ELBW or very preterm in Victoria, Australia.	Probable DCD (performance‐based tests)	20	190	NR (8–9)	NR (8–9)	75.0	40.5	No (not assessed)	None	BASC‐parent: internalising subscale (parent‐rated internalising); BASC‐teacher rated (teacher‐rated internalising)
Dewey et al.	2002	Population screening: public and private schools in Calgary, Canada.	Probable DCD (performance‐based tests, screening questionnaires)	45	78	11.8 (NR)	11.4 (NR)	55.7	75.6	No (measured, not excluded)	None	CBCL – internalising (parent‐rated internalising)
Francis & Piek	2003	Population screening: Grades 3–5 in primary schools in Perth, Australia.	Probable DCD (performance‐based test)	42	42	NR (7–11)	NR (7–11)	52	52	No (not assessed)	Age and gender	CDI (self‐rated depression)
Hill & Brown	2013	Convenience sample: DCD adults via support groups and higher education institutions. TD adults via higher education institutions and local community centres across UK.	Confirmed DCD (medical report)	36	49	29.28 (19–59)	27.84 (18–56)	42	49	Yes (self‐report)	None	STAI‐Trait (self‐rated anxiety); STAI‐State (self‐rated anxiety); BDI (self‐rated depression)
King‐Dowling et al.	2015	Population screening: community organizations in Southern Ontario, Canada.	Probable DCD (performance‐based tests)	37	117	4.92 (NR)	4.92 (NR)	78	42	No (not assessed)	None	CBCL (parent‐rated internalising)
Li et al.	2018	Population screening: Participants from the Physical Health and Activity Study Team project from schools in Canada.	Probable DCD (performance‐based test)	79	1,127	13.45 (12–14)	13.40 (12–14)	38.0	51.6	No (not assessed)	None	Kessler‐6 Scale (self‐rated internalising)
Missiuna et al.	2014	Population screening: Grades 4–8 from schools within two health boards regions in Canada.	Confirmed DCD (researcher assessment)	68	91	11.8 (NR)	12.0 (NR)	60	51	Yes (parent‐/school‐report)	None	CDI (self‐rated depression); CDI (parent‐rated depression); SCARED (self‐rated anxiety); SCARED (parent‐rated anxiety)
Piek et al.	2007	Screening of a twin sample: Monozygotic twins discordant for DCD on the Australian Twin Registry.	Probable DCD (screening questionnaire)	24	24	11.91 (6.45–16.99)	11.91 (6.45–16.99)	45.8	45.8	Yes (screening measure)	Age, gender, (also genes and shared environmental factors)	Twin and Sibling Questionnaire – Sad Affect subscale (parent‐rated depression)
Piek et al.	2008	Population screening: Kindergarten children in a primary school in Western Australia.	Probable DCD (performance‐based tests)	14	26	NR; Total sample mean = 4.3 (3.75–5.33)	NR; Total sample mean = 4.3 (3.75–5.33)	71.4	NR	No (not assessed)	None^d^	CBCL – anxious/depressed subscale (parent‐rated internalising)
Pratt & Hill	2011	Convenience sample: DCD from support groups, existing research, schools within London and south‐east England; TD from existing research and schools within London and south‐east England.	Confirmed DCD (medical report)	27	35	10.08 (6–15)	9.38 (6–15)	74.1	51.4	Yes (medical report and/or screening measure)	None	SCAS‐Parent (parent‐rated anxiety)
Schoemaker & Kalverboer	1994	Population screening: Dutch mainstream schools.	Probable DCD (performance‐based test)	18	18	7.3 (6.1–9.0)	7.3 (6.0–9.1)	83.3	83.3	No (not assessed)	Age, gender	STAIC‐Trait (self‐rated anxiety); STAIC‐State (self‐rated anxiety)
Skinner & Piek (child sample)	2001	Population screening: Primary schools in Western Australia.	Probable DCD (performance‐based tests)	58	58	NR (8–10)	NR (8–10)	31	31	No (not assessed)	Age, gender	STAIC‐Trait (self‐rated anxiety); STAIC‐State (self‐rated anxiety)
Skinner & Piek (adolescent sample)	2001	Population screening: High schools in Western Australia.	Probable DCD (performance‐based tests)	51	51	NR (12–14)	NR (12–14)	43	43	No (not assessed)	Age, gender	STAIC‐Trait (self‐rated anxiety); STAIC‐State (self‐rated anxiety)
van den Huevel et al.	2016	Population screening: Primary schools in middle and eastern Netherlands.	Confirmed DCD (researcher assessment)	TRF: 20 SDQ 22	TRF: 307; SDQ: 339	NR For total sample (not all included in analysis): 7.0 (4.3–10.3)	NR For total sample (not all included in analysis): 7.2 (4.0–10.8)	NR For total sample (not all included in analysis): 69.6	NR For total sample (not all included in analysis): 49.1	No (measured, not excluded)	None	TRF – internalising subscale (teacher‐rated internalising) SDQ‐teacher‐ Emotional subscale (teacher‐rated internalising)
Wagner et al.	2012	DCD: Clinical sample from occupational therapy groups in Germany TD: elementary schools in Germany.	Confirmed DCD (researcher assessed)	35	35	7.69 (5–11)	NR (reported to be matched)	77.1	77.1	No (not assessed)	Age, gender	IDS – Internalising scale (parent‐rated internalising)
Watson & Knott	2006	DCD: Clinical sample from Occupational therapy department in West of Scotland TD: Primary schools in West of Scotland.	Confirmed DCD (medical report)	15	30	10.2 (8–12)	10.25 (NR)	80	80	Yes (self‐report or medical report)	Age and gender	Birleson Depression Measure (self‐rated depression)

BASC, Behaviour Assessment System for Children; BDI, Beck Depression Inventory; CBCL, Child Behaviour Checklist; CDI, Children's Depression Inventory; DCD, Developmental Coordination Disorder; ELBW, Extremely Low Birth Weight; IDS, Intelligence and Developmental Scales; NR, Not Reported; SCARED, Screen for Child Anxiety Related Disorders; SCAS, Spence Children's Anxiety Scale; SDQ, Strengths and Difficulties Questionnaire; SMFQ, Short Moods and Feelings Questionnaire; STAI, State‐Trait Anxiety Inventory for Adults; STAIC, State‐Trait Anxiety Inventory for Children; TD, Typically Developing; TRF, Teacher Report Form.

a Additional confounders included in adjusted analysis: maternal depression, family adversity, IQ, social communication.

b Based on reported age for combined male and female subsamples.

c Study reports two alternative performance‐based measures for determining DCD. Criteria based on the MABC were used for comparability with other studies.

d Adjusted for gender and IQ, but unadjusted data used in meta‐analysis.

Sixteen studies identified individuals with and without DCD/probable DCD via population‐based screening of community samples (Campbell, Missiuna, & Vaillancourt, 2012; Chen, Tseng, Hu, & Cermak, 2009; Dewey et al., 2002; Francis & Piek, 2003; Harrowell et al., 2017; van den Heuvel, Jansen, Reijneveld, Flapper, & Smits‐Engelsman, 2016; King‐Dowling et al., 2015; Li et al., 2018; Missiuna et al., 2014; Piek, Bradbury, Elsley, & Tate, 2008; Schoemaker & Kalverboer, 1994; Skinner & Piek, 2001; Wagner et al., 2016). Of the remaining studies, five recruited DCD participants through selective or convenience samples such as clinical referrals or support groups (Crane, Sumner, & Hill, 2017; Hill & Brown, 2013; Pratt & Hill, 2011; Wagner, Bös, Jascenoka, Jekauc, & Petermann, 2012; Watson & Knott, 2006), one recruited participants through screening a clinical population of children born with extremely low birth weight (Davis et al., 2007), and one sampled from a monozygotic twin population (Piek et al., 2007).

The studies varied in their operationalisation of the DCD group. Ten studies confirmed a DCD diagnosis via independent assessment of diagnostic criteria (Chen et al., 2009; Harrowell et al., 2017; van den Heuvel et al., 2016; Missiuna et al., 2014; Wagner et al., 2012) or via clinical reports (Crane et al., 2017; Hill & Brown, 2013; Pratt & Hill, 2011; Watson & Knott, 2006). Thirteen studies identified those as probable DCD based on performance‐based tests of motor function (Davis et al., 2007; Dewey et al., 2002; Francis & Piek, 2003; King‐Dowling et al., 2015; Li et al., 2018; Piek et al., 2008; Schoemaker & Kalverboer, 1994; Skinner & Piek, 2001; Wagner et al., 2016) or by parent‐report questionnaires (Campbell et al., 2012; Piek et al., 2007).

Most studies recruited children and adolescents, with only one study conducted with adults (Hill & Brown, 2013). Of the child and adolescent studies, ten included adolescents aged 12 or over (Dewey et al., 2002; Harrowell et al., 2017; Li et al., 2018; Missiuna et al., 2014; Piek et al., 2007; Pratt & Hill, 2011; Skinner & Piek, 2001; adolescent sample; Wagner et al., 2016; Watson & Knott, 2006). There was a mix of male and female participants across the studies, with fourteen having majority male participants (Campbell et al., 2012; male sample; Crane et al., 2017; Davis et al., 2007; Dewey et al., 2002; Francis & Piek, 2003; Harrowell et al., 2017; male sample; King‐Dowling et al., 2015; Missiuna et al., 2014; Piek et al., 2008; Pratt & Hill, 2011; Schoemaker & Kalverboer, 1994; van den Heuvel et al., 2016; Wagner et al., 2012; Watson & Knott, 2006). Six studies explicitly excluded DCD participants with ADHD based on self‐/parent‐/school‐reported diagnoses (Crane et al., 2017; Hill & Brown, 2013; Missiuna et al., 2014; Watson & Knott, 2006) or based on scores on standardised screening questionnaires (Piek et al., 2007) or either of the two (Pratt & Hill, 2011). The remaining studies either did not assess for ADHD (Campbell et al., 2012; Davis et al., 2007; Francis & Piek, 2003; King‐Dowling et al., 2015; Li et al., 2018; Piek et al., 2008; Schoemaker & Kalverboer, 1994; Skinner & Piek, 2001; Wagner et al., 2012) or measured symptoms but did not exclude (Chen et al., 2009; Dewey et al., 2002; Harrowell et al., 2017; van den Heuvel et al., 2016; Wagner et al., 2016).

All outcome measures of internalising symptoms were based on questionnaires. Most studies measured overall internalising symptoms using the Child Behaviour Checklist (Chen et al., 2009; Dewey et al., 2002; King‐Dowling et al., 2015), Strengths and Difficulties Questionnaire (Crane et al., 2017; Harrowell et al., 2017; van den Heuvel et al., 2016; Wagner et al., 2016), Behaviour Assessment System for Children (BASC; Davis et al., 2007), Teacher Report Form (van den Heuvel et al., 2016), Kessler‐6 (Li et al., 2018), or the Intelligence and Developmental Scales (Wagner et al., 2012). Nine studies specifically measured depressive symptoms using the Children's Depression Inventory (Francis & Piek, 2003; Missiuna et al., 2014), Beck Depression Inventory (Hill & Brown, 2013), Short Mood and Feelings Questionnaire (Harrowell et al., 2017), BASC – Depression subscale (Campbell et al., 2012), Twin and Sibling Questionnaire – Sad Affect subscale (Piek et al., 2007), or the Birleson Depression Measure (Watson & Knott, 2006). Six studies specifically measured levels of anxiety symptoms using the State‐Trait Anxiety Inventory for Children (Schoemaker & Kalverboer, 1994; Skinner & Piek, 2001), State‐Trait Anxiety Inventory for Adults (Hill & Brown, 2013), Screen for Child Anxiety Related Disorders (Missiuna et al., 2014), or Spence Children's Anxiety Scale (Pratt & Hill, 2011). Overall, the outcome measures were based on parent‐report in seven studies (Chen et al., 2009; Dewey et al., 2002; King‐Dowling et al., 2015; Piek et al., 2007, 2008; Pratt & Hill, 2011; Wagner et al., 2012, 2016), teacher‐report in two studies (Crane et al., 2017; van den Heuvel et al., 2016), self‐report in eleven studies (Campbell et al., 2012; Francis & Piek, 2003; Harrowell et al., 2017; Hill & Brown, 2013; Li et al., 2018; Schoemaker & Kalverboer, 1994; Skinner & Piek, 2001; Watson & Knott, 2006) and a combination in two studies (Davis et al., 2007; Missiuna et al., 2014).

### Risk of bias

The risk of bias in the included studies is summarised in Table 2. Selection bias was variable. The use of a population screening method in 16 studies ensured the DCD sample was somewhat representative of the population studied. However, seven studies recruited the DCD sample from a selective group or convenience sample, which may be at greater risk of bias. This included children born very preterm or with extremely low birth weight (Davis et al., 2007), volunteer samples from DCD support groups (Crane et al., 2017; Hill & Brown, 2013; Pratt & Hill, 2011), monozygotic twin samples (Piek et al., 2007), or clinical samples from occupational therapy services (Wagner et al., 2012; Watson & Knott, 2006). In most studies, the TD group was drawn from the same community (e.g. school, geographical location) as the DCD group, except for four studies that were from a different source (Crane et al., 2017; Hill & Brown, 2013; Pratt & Hill, 2011; Wagner et al., 2012) and thus had an increased risk from selection bias. Additionally, while 10 studies confirmed diagnoses of DCD by independent assessment or clinical reports (Chen et al., 2009; Crane et al., 2017; Harrowell et al., 2017; van den Heuvel et al., 2016; Hill & Brown, 2013; Missiuna et al., 2014; Pratt & Hill, 2011; Wagner, 2017; Watson & Knott, 2006), 13 did not confirm key diagnostic criteria (Campbell et al., 2012; Davis et al., 2007; Dewey et al., 2002; Francis & Piek, 2003; King‐Dowling et al., 2015; Li et al., 2018; Piek et al., 2007, 2008; Schoemaker & Kalverboer, 1994; Skinner & Piek, 2001; Wagner et al., 2016). Caution should be taken, therefore, when attributing differences in internalising symptoms in these studies to DCD. Most studies were cross‐sectional and, therefore, unable to account for internalising symptoms prior to the development of motor difficulties. Of the longitudinal studies, one controlled for baseline internalising symptoms (Wagner et al., 2016).

**Table 2:** Summary of risk of bias

Study	DCD sample representative of population	Typically developing sample from the same population	DCD diagnosis confirmed	Prior internalising symptoms controlled for	Controls for gender	Controls for age	Standardised outcome measure	Sufficient length of follow‐up	Sufficient follow‐up rate
Campbell et al. (2012, male)	✔	✔			✔	✔	✔		
Campbell et al. (2012, female)	✔	✔			✔	✔	✔		
Chen et al. (2009)	✔	✔	✔				✔		
Crane et al. (2017)			✔				✔		
Davis et al. (2007)		✔					✔		
Dewey et al. (2002)	✔	✔					✔		
Francis and Piek (2003)	✔	✔			✔	✔	✔		
Harrowell et al. (2017, male)	✔	✔	✔		✔	✔	✔	✔	
Harrowell et al. (2017, female)	✔	✔	✔		✔	✔	✔	✔	
Hill and Brown (2013)			✔				✔		
King‐Dowling et al. (2015)	✔	✔					✔		
Li et al. (2018)	✔	✔					✔		
Missiuna et al. (2014)	✔	✔	✔				✔		
Piek et al. (2007)		✔			✔	✔	✔		
Piek et al. (2008)	✔	✔					✔		
Pratt and Hill (2011)			✔				✔		
Schoemaker and Kalverboer (1994)	✔	✔			✔	✔	✔		
Skinner and Piek (2001, adolescent)	✔	✔			✔	✔	✔		
Skinner and Piek (2001, child)	✔	✔			✔	✔	✔		
van den Heuvel et al. (2016)	✔	✔	✔				✔		
Wagner et al. (2012)			✔		✔	✔	✔		
Wagner et al. (2016)	✔	✔		✔	✔	✔	✔	✔	
Watson and Knott (2006)		✔	✔		✔	✔	✔		

The studies varied in the comparability of study groups and control for important confounders. Age and gender were controlled in 11 studies through either matched groups (Campbell et al., 2012; Francis & Piek, 2003; Piek et al., 2007; Schoemaker & Kalverboer, 1994; Skinner & Piek, 2001; Wagner et al., 2012; Watson & Knott, 2006) or adjusted analyses (Harrowell et al., 2017; Wagner et al., 2016). The study by Piek et al. (2007) adopted a monozygotic twin design which also controls for a wide range of genetic and shared environmental factors. The remaining studies failed to sufficiently control for age and gender. Although one such study did adjust for differences in gender in the analysis (Piek et al., 2008), it also included intellectual ability as a covariate, and so the unadjusted difference between the groups was used in the meta‐analysis to ensure comparability (Voils et al., 2011).

All studies utilised outcome measures with established validity and reliability psychometrics. It should be noted that three studies only reported specific narrow‐band depression or anxiety subscales, as opposed to using the broad‐band internalising scales (Campbell et al., 2012; Piek et al., 2008). These might raise concerns around selective reporting.

Finally, only three studies included a longitudinal follow‐up (Harrowell et al., 2017; male & female sample; Wagner et al., 2016). This was over a year in all three studies. However, there were high rates of attrition in all three.

### Internalising symptoms

**Figure 2:**
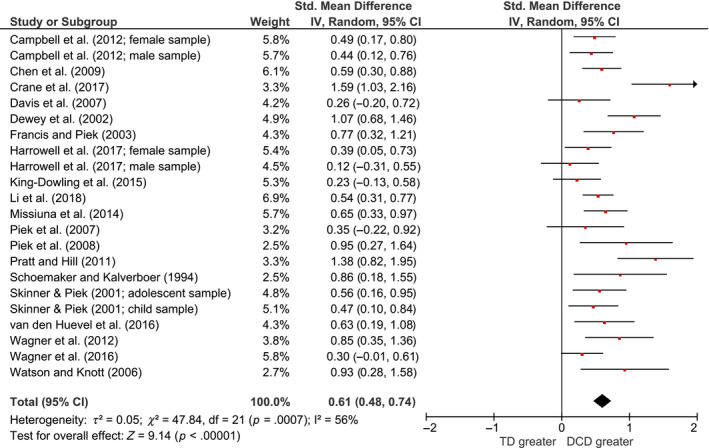
Forest plot for total internalising symptoms across all studies [Colour figure can be viewed at wileyonlinelibrary.com]

Since only one study based on an adult sample was identified, that study was excluded from the remaining analyses. Across the 22 studies with children and adolescents, those with DCD or probable DCD were found to have higher levels of internalising symptoms than TD controls with a medium effect size (*g *=* *0.61; 95% CI: 0.48–0.74; see Figure 2 for forest plot). There was significant moderate heterogeneity among the studies (*I* ^2^ = 56%; χ^2^ = 47.84; *p *=* *.0007).

### Moderator analysis

The results of the moderator analyses are summarised in Table 3. The results revealed that the effect size was significantly larger in studies that utilised a cross‐sectional design (vs. longitudinal), that included a majority male sample in the DCD group (vs. majority female) and that recruited a selective or convenience sample of participants (as opposed to population‐based screening). There was also a trend (*p *=* *.05) towards a greater effect size in studies that did not control for important confounders and in studies that excluded individuals with a diagnosis of ADHD. No significant effect was found for age, confirmation of DCD diagnosis, outcome respondent or type of internalising measure.

**Table 3:** Summary of results for moderators

Moderator	*k*	Total *n*	*g*	95% CI	*Q*	*p*
Design					9.33	.002
Longitudinal	3	937	0.29	0.09–0.49		
Cross‐sectional	20	3,645	0.67	0.54–0.80		
Age					0.02	.88
Included adolescents	10	2,682	0.60	0.39–0.80		
Not included adolescents	12	1,780	0.62	0.44–0.79		
Gender					4.72	.03
>50% male	15	1,889	0.71	0.52–0.91		
>50% female	7	2,573	0.46	0.33–0.58		
Confirmed diagnoses					2.30	.13
Confirmed DCD	9	998	0.75	0.48–1.01		
Probable DCD	13	3,434	0.52	0.39–0.65		
Population‐based design					5.06	0.02
Population screening	17	4,176	0.52	0.41–0.62		
Selective sample	4	286	1.02	0.59–1.45		
ADHD					3.70	.05
Excluded ADHD	5	379	0.97	0.53–1.41		
Did not exclude ADHD	17	4,083	0.52	0.41–0.63		
Controlled for confounding					3.81	0.05
Age and gender controlled	12	1,752	0.48	0.36–0.60		
Age and gender not controlled	10	2,710	0.74	0.51–0.97		
Outcome respondent^a^					2.18	.14
Self‐report	10	1,907	0.50	0.39–0.62		
Parent/teacher‐report	12	2,396	0.71	0.46–0.97		
Outcome measure type					1.13	.57
Total internalising	12	3,492	0.59	0.39–0.78		
Depression only	5	495	0.54	0.36–0.72		
Anxiety only	4	316	0.77	0.38–1.16		
Sensitivity analyses						
Study quality					4.42	.04
High quality	10	1,638	0.46	0.34–0.58		
Low quality	12	2,800	0.72	0.51–0.93		

ADHD, Attention‐Deficit Hyperactivity Disorder; DCD, developmental coordination disorder.

a Missiuna et al. (2014) excluded from analysis due to using both self‐ and observer‐report. Inclusion of each type of measure from this study, independently, did not significantly change the results.

### Sensitivity analysis

A sensitivity analysis was conducted to include only those studies meeting five or more criteria on the NOS. Moderator analysis identified a significant difference between the high‐quality and low‐quality studies (*Q *=* *4.42; *p *=* *.04). Analysis of the higher quality studies (*k *=* *10) found a smaller, but still moderate, effect of DCD on internalising symptoms (*g = *0.46; 95% CI: 0.34–0.58). There was also no evidence of significant heterogeneity among these ten studies (*Q *=* *5.95; *p *=* *.55; *I* ^2 ^= 0%).

### Publication bias

The funnel plot (see Figure S1) displayed some asymmetry, with smaller studies tending to report larger effect sizes, possibly indicative of publication bias. Eggers test was statistically significant, supporting the presence of publication bias (Egger's bias = 2.38; 95% CI: 0.20–4.56; *p *=* *.02). However, Duval and Tweedie's trim and fill procedure did not impute additional studies and, therefore, the effect size adjusted for publication bias was identical to the nonadjusted effect size. Rosenthal's Fail‐safe *N*, suggested that the number of studies with null results that would have to be included to produce a nonsignificant combined effect size is 1,076. This is substantially larger than the minimum required when applying Rosenthal's (1979) formula (i.e. 120).

## Discussion

The present systematic review and meta‐analysis indicate that children and adolescents with DCD or probable DCD experience greater levels of internalising symptoms compared to their TD peers. The magnitude of this difference suggests a moderate effect size, with individuals with DCD scoring over half a standard deviation higher. This moderate effect, although reduced slightly, remained robust after excluding lower quality studies. Methodological and participant factors that may moderate the magnitude of this effect have also been identified.

### DCD and internalising symptoms

The findings are in line with the emerging consensus that DCD can have a significant impact on an individual's mental health (Caçola, 2016; Mancini et al., 2016, 2018; Missiuna & Campbell, 2014). Notably, the magnitude of the effect identified is comparable, if not greater, than that found in meta‐analyses of a wide range of chronic physical health conditions (Pinquart & Shen, 2011a, 2011b).

The environmental stress hypothesis is a framework that was introduced to account for the relationship between DCD and mental health (Cairney, Rigoli, & Piek, 2013). It suggests that the motor impairments in DCD can expose an individual to a variety of secondary stressors, which over time can lead to poorer mental health. Although potential mediators were not explored in this review, they have been outlined previously (Mancini et al., 2016, 2018). They include peer victimisation (Campbell et al., 2012), reduced leisure activities (Raz‐Silbiger et al., 2015), impaired social skills (Wilson, Piek et al., 2013), poorer self‐esteem (Rigoli et al., 2012), physical inactivity (Li et al., 2018), reduced social support (Rigoli et al., 2017) and lower perceived academic competence (Lingam et al., 2012). Individuals with DCD may also experience impairments to various cognitive abilities, including executive function (Wilson, Ruddock, Smits‐Engelsman, Polatajko, & Blank, 2013) and social cognition (Cummins, Piek, & Dyck, 2005). This may further impact on self‐regulation and mental well‐being (Lantrip, Isquith, Koven, Welsh, & Roth, 2016; Letkiewicz et al., 2014). Future meta‐analyses regarding the magnitude of the effects for these potential mediators will be important, providing further opportunities for intervention.

### Moderating factors

The present review identified several methodological factors that might moderate the degree to which DCD is associated with internalising symptoms. As expected, the effect size was likely overestimated in cross‐sectional compared to longitudinal studies, and in convenience or clinic‐referred samples compared to samples recruited via population‐based screening. Such methodologies have less control of confounding factors and a less representative selection of DCD participants (e.g. more severe impairments in clinical samples). There was also a trend towards larger effect sizes in studies that failed to control for age and gender. Again, the magnitude of the effect sizes in these studies was likely inflated by confounding factors (Deeks et al., 2003). No significant effect was found for the DCD criteria used. This suggests that, although establishing all DCD diagnostic criteria is important for the quality of research in this area (Zwicker et al., 2013), failing to do this might not have a substantial impact on the results. This may be because it is specifically the motor difficulties, as tested by screening measures in studies of ‘probable DCD’, which affect internalising symptoms, rather than issues surrounding having a diagnosis. Population‐based screening, longitudinal design and control for confounders should therefore take priority in future studies.

Participant factors that might moderate the effect of DCD on internalising symptoms were also identified. The effect was larger in studies with a majority male sample. This would suggest that DCD has a greater impact on the mental health of males and is in line with the findings of Sigurdsson et al. (2002). This is particularly important given the prevalence of DCD may be greater in males (Kirby & Sugden, 2007; Missiuna, 1994). It has been suggested that male children attribute greater value to physical activity and sports compared to females, which might account for the larger impact of DCD on their well‐being (Poulsen, Ziviani, & Cuskelly, 2006; Poulsen, Ziviani, Cuskelly, & Smith, 2007). However, although significant, the difference in the magnitude of the effect sizes was minimal. Additionally, only two of the included studies reported within‐study comparisons of the impact of DCD on males and females, with one suggesting no difference (Campbell et al., 2012) and the other suggesting a greater impact for females (Harrowell et al., 2017). Regardless of which gender experiences the larger effect, there is evidence that DCD can impact on the mental health of both genders and perhaps it is the mechanism by which this occurs that differs (Li et al., 2018).

There was also a trend towards larger effect sizes in studies that specifically excluded participants with ADHD. This contradicts what might have been expected from previous research (Martin, Piek, & Hay, 2006; Missiuna et al., 2014; Rasmussen & Gillberg, 2000). One possible explanation for this finding is that children with comorbid ADHD are more likely to be diagnosed and to subsequently receive support for their difficulties (Heath, Toste, & Missiuna, 2005; Rivard, Missiuna, Hanna, & Wishart, 2011). However, it should also be noted that there were only five studies included in the meta‐analysis that specifically excluded participants with ADHD. All five studies were also of a lower quality. It is a more plausible explanation that methodological limitations inflated their combined effect size.

Contrary to what might be expected (Missiuna, Moll, King, King, & Law, 2007; Skinner & Piek, 2001), no significant moderating effect was found for age. However, only one study included in the present review reported separate outcomes for adolescents and children. The moderator categories for the meta‐analysis were based on somewhat arbitrary criteria (i.e. studies that included adolescents in their sample, as opposed to studies with a pure adolescent sample) which may have prevented the detection of differences between the age groups.

Finally, no significant effect was found for the type of outcome measure or respondent, suggesting that DCD may be associated with elevated levels of depression, anxiety and overall internalising symptoms, regardless of the person rating it. However, there are limited within‐study comparisons available despite previous research highlighting variability between parent‐ and self‐reported internalising symptoms (Cantwell et al., 1997). As such, collecting information from multiple informants will maximise reliability in future research and clinical practice.

### Strengths and limitations

There are several limitations of this review. First, the quality of the included studies was variable. Most studies were based only on cross‐sectional data, which make it difficult to establish causality. Although three longitudinal studies were included, only one controlled for baseline measures of internalising symptoms and all reported a high rate of attrition. Many of the studies also failed to control for important confounders (i.e. age and gender) and to establish all DCD diagnostic criteria. The review also focused on studies that dichotomised participants into DCD and TD groups, whereas motor coordination can be understood as a continuum of ability. This dichotomy can miss the variation in motor skills that exists within each group, as well as changes over time and across different measures.

The moderator analysis should also be interpreted with caution. As outlined above, there were an insufficient number of studies within some of the moderator categories to reliably explore their impact (including age and comorbid ADHD). It is of note that many studies failed to measure ADHD symptomatology, despite evidence for high rates of comorbidity (Martin et al., 2006). Most of the studies were also conducted in western, developed countries and, therefore, generalisation to other countries is limited. Additionally, only one study with adults was identified; therefore, the extent to which elevated internalising symptoms persist into adulthood is unclear.

However, this review is the first attempt to systematically synthesise the evidence on internalising symptoms in individuals with DCD and provide a pooled summary of the effect size. Publication bias is unlikely to have a substantial impact. Additionally, despite methodological limitations of the included studies, potential moderating factors have been identified. The effect size also remained substantial, and heterogeneity reduced, after excluding lower quality studies.

## Implications and conclusion

The findings have important clinical and research implications. It can be concluded that individuals with DCD have an increased risk of developing elevated levels of internalising symptoms. The difference of half a standard deviation between individuals with DCD and their peers could be considered clinically important (Norman, Sloan, & Wyrwich, 2003). This would support the practice of routine screening of mental health difficulties in individuals with DCD and motor impairment. Given that DCD is poorly understood among professionals (Gaines et al., 2008; Wilson, Neil et al., 2013) and that families often report difficulties obtaining support (Stephenson & Chesson, 2008), such routine screening could be useful across a range of services (including schools, occupational therapy, physical healthcare and mental healthcare). The findings also highlight the need for professionals in mental health services to be aware of the disorder and how it impacts their patients. Additionally, the findings support the need for the development of psychosocial interventions for DCD with a focus on the secondary stressors that might mediate the link between motor difficulties and emotional well‐being (Missiuna et al., 2012).

Future research should focus on high‐quality longitudinal studies to better understand the causal link between DCD and internalising symptoms, including the role of important mediators. It is recommended that studies include probability sampling strategies and control for confounders and the stability of internalising symptoms over time. This review has also highlighted the need for more research investigating mental health in adults with DCD, especially given that the impact of DCD has been found to continue into adulthood (Cousins & Smyth, 2003; Hill et al., 2011; Kirby, Williams, Thomas, & Hill, 2013). Research investigating the effectiveness of routine screening for mental health difficulties in DCD and psychosocial interventions would also provide insight into the improved management of DCD. Given the major economic impact of poor mental health (Trautmann, Rehm, & Wittchen, 2016) and the increasing focus on improving psychological well‐being in government policy (Department of Health, 2011), the need to identify and support those individuals most at risk of mental health difficulties is crucial.

**Key points**

- Research indicates that individuals with DCD may be at a greater risk of mental health difficulties. However, there has been no attempt to systematically consolidate this research.
- This meta‐analysis suggests that children and adolescents with DCD experience greater levels of internalising symptoms (i.e. depression and anxiety) than their typically developing peers.
- This highlights the potential need for routine screening of mental health difficulties in individuals with DCD.
- It also highlights the need for psychosocial interventions for this population.

## Supporting information


**Appendix S1.** Summary of search terms used.
**Figure S1.** Funnel plot of effect sizes and standard error.

## References

[jcpp13001-bib-0001] American Psychiatric Association (2013). Diagnostic and statistical manual of mental disorders (5th edn). Washington, DC: American Psychiatric Association.

[jcpp13001-bib-0002] Blank, R. , Smits‐Engelsman, B. , Polatajko, H. , & Wilson, P. (2012). European Academy for Childhood Disability (EACD): Recommendations on the definition, diagnosis and intervention of developmental coordination disorder (long version). Developmental Medicine & Child Neurology, 54, 54–93. 22171930 10.1111/j.1469-8749.2011.04171.x

[jcpp13001-bib-0003] Borenstein, M. , Hedges, L.V. , Higgins, J.P.T. , & Rothstein, H.R. (2009). Introduction to meta‐analysis. West Sussex, UK: Wiley.

[jcpp13001-bib-0004] Caçola, P. (2016). Physical and mental health of children with developmental coordination disorder. Frontiers in Public Health, 4, 224. 27822464 10.3389/fpubh.2016.00224 PMC5075567

[jcpp13001-bib-0005] Cairney, J. , Rigoli, D. , & Piek, J. (2013). Developmental coordination disorder and internalizing problems in children: The environmental stress hypothesis elaborated. Developmental Review, 33, 224–238.

[jcpp13001-bib-0006] Campbell, W.N. , Missiuna, C. , & Vaillancourt, T. (2012). Peer victimization and depression in children with and without motor coordination difficulties. Psychology in the Schools, 49, 328–341.

[jcpp13001-bib-0007] Cantwell, D.P. , Lewinsohn, P.M. , Rohde, P. , & Seeley, J.R. (1997). Correspondence between adolescent report and parent report of psychiatric diagnostic data. Journal of the American Academy of Child and Adolescent Psychiatry, 36, 610–619. 9136495 10.1097/00004583-199705000-00011

[jcpp13001-bib-0008] Chen, Y.W. , Tseng, M.H. , Hu, F.C. , & Cermak, S.A. (2009). Psychosocial adjustment and attention in children with developmental coordination disorder using different motor tests. Research in Developmental Disabilities, 30, 1367–1377. 19625163 10.1016/j.ridd.2009.06.004

[jcpp13001-bib-0009] Cousins, M. , & Smyth, M.M. (2003). Developmental coordination impairments in adulthood. Human Movement Science, 22, 433–459. 14624827 10.1016/j.humov.2003.09.003

[jcpp13001-bib-0010] Crane, L. , Sumner, E. , & Hill, E.L. (2017). Emotional and behavioural problems in children with developmental coordination disorder: Exploring parent and teacher reports. Research in Developmental Disabilities, 70, 67–74. 28915470 10.1016/j.ridd.2017.08.001

[jcpp13001-bib-0011] Cummins, A. , Piek, J.P. , & Dyck, M.J. (2005). Motor coordination, empathy, and social behaviour in school‐aged children. Developmental Medicine and Child Neurology, 47, 437–442. 15991862 10.1017/s001216220500085x

[jcpp13001-bib-0012] Damme, T.V. , Simons, J. , Sabbe, B. , & van West, D. (2015). Motor abilities of children and adolescents with a psychiatric condition: A systematic literature review. World Journal of Psychiatry, 5, 315–329. 26425445 10.5498/wjp.v5.i3.315 PMC4582307

[jcpp13001-bib-0013] Davis, N.M. , Ford, G.W. , Anderson, P.J. , & Doyle, L.W. (2007). Developmental coordination disorder at 8 years of age in a regional cohort of extremely‐low‐birthweight or very preterm infants. Developmental Medicine and Child Neurology, 49, 325–330. 17489804 10.1111/j.1469-8749.2007.00325.x

[jcpp13001-bib-0014] Deeks, J.J. , Dinnes, J. , D'Amico, R. , Sowden, A.J. , Sakarovitch, C. , Song, F. , … & Altman, D. (2003). Evaluating non‐randomised intervention studies. Health Technology Assessment, 7, 1–179. 10.3310/hta7270 14499048

[jcpp13001-bib-0015] Department of Health . (2011). No health without mental health: A cross‐government mental health outcomes strategy for people of all ages. London: Department of Health.

[jcpp13001-bib-0016] Dewey, D. , Kaplan, B.J. , Crawford, S.G. , & Wilson, B.N. (2002). Developmental coordination disorder: Associated problems in attention, learning, and psychosocial adjustment. Human Movement Science, 21, 905–918. 12620725 10.1016/s0167-9457(02)00163-x

[jcpp13001-bib-0017] Duval, S. , & Tweedie, R. (2000). Trim and fill: A simple funnel‐plot–based method of testing and adjusting for publication bias in meta‐analysis. Biometrics, 56, 455–463. 10877304 10.1111/j.0006-341x.2000.00455.x

[jcpp13001-bib-0018] Francis, M. , & Piek, J. (2003) The effects of perceived social support and self‐worth on depressive symptomatology in children with and without developmental coordination disorder (DCD). Paper presented at the 38th APS Annual Conference, Perth, Western Australia.

[jcpp13001-bib-0019] Gaines, R. , Missiuna, C. , Egan, M. , & McLean, J. (2008). Educational outreach and collaborative care enhances physician's perceived knowledge about developmental coordination disorder. BMC Health Services Research, 8, 21. 18218082 10.1186/1472-6963-8-21 PMC2254381

[jcpp13001-bib-0020] Harrowell, I. , Hollén, L. , Lingam, R. , & Emond, A. (2017). Mental health outcomes of developmental coordination disorder in late adolescence. Developmental Medicine and Child Neurology, 59, 973–979. 28512766 10.1111/dmcn.13469 PMC5573907

[jcpp13001-bib-0021] Hedges, L. , & Olkin, I. (1985). Statistical models for meta‐analysis. San Diego, CA: Academic Press.

[jcpp13001-bib-0022] Heath, N.L. , Toste, J.R. , & Missiuna, C. (2005). An exploration of the relationship between motor impairment and emotional/behavioural difficulties amongst children suspected of having DCD. Israeli Journal of Occupational Therapy, 14, 153–170.

[jcpp13001-bib-0023] van den Heuvel, M. , Jansen, D.E.M.C. , Reijneveld, S.A. , Flapper, B.C.T. , & Smits‐Engelsman, B.C.M. (2016). Identification of emotional and behavioral problems by teachers in children with developmental coordination disorder in the school community. Research in Developmental Disabilities, 51–52, 40–48. 10.1016/j.ridd.2016.01.008 26780353

[jcpp13001-bib-0024] Hill, E.L. , & Brown, D. (2013). Mood impairments in adults previously diagnosed with developmental coordination disorder. Journal of Mental Health, 22, 334–340. 23323694 10.3109/09638237.2012.745187

[jcpp13001-bib-0025] Hill, E.L. , Brown, D. , & Sorgardt, K.S. (2011). A preliminary investigation of quality of life satisfaction reports in emerging adults with and without developmental coordination disorder. Journal of Adult Development, 18, 130–134.

[jcpp13001-bib-0026] King‐Dowling, S. , Missiuna, C. , Rodriguez, M.C. , Greenway, M. , & Cairney, J. (2015). Co‐occurring motor, language and emotional–behavioral problems in children 3–6 years of age. Human Movement Science, 39, 101–108. 25436914 10.1016/j.humov.2014.10.010

[jcpp13001-bib-0027] Kirby, A. , & Sugden, D.A. (2007). Children with developmental coordination disorders. Journal of the Royal Society of Medicine, 100, 182–186. 17404341 10.1258/jrsm.100.4.182 PMC1847727

[jcpp13001-bib-0028] Kirby, A. , Williams, N. , Thomas, M. , & Hill, E.L. (2013). Self‐reported mood, general health, wellbeing and employment status in adults with suspected DCD. Research in Developmental Disabilities, 34, 1357–1364. 23417140 10.1016/j.ridd.2013.01.003

[jcpp13001-bib-0029] Lantrip, C. , Isquith, P.K. , Koven, N.S. , Welsh, K. , & Roth, R.M. (2016). Executive function and emotion regulation strategy use in adolescents. Applied Neuropsychology: Child, 5, 50–55. 25650638 10.1080/21622965.2014.960567

[jcpp13001-bib-0030] Letkiewicz, A.M. , Miller, G.A. , Crocker, L.D. , Warren, S.L. , Infantolino, Z.P. , Mimnaugh, K.J. , & Heller, W. (2014). Executive function deficits in daily life prospectively predict increases in depressive symptoms. Cognitive Therapy and Research, 38, 612–620. 10.1007/s10608-014-9629-5 PMC10544784 37786427

[jcpp13001-bib-0031] Li, Y.C. , Kwan, M.Y. , Clark, H.J. , Hay, J. , Faught, B.E. , & Cairney, J. (2018). A test of the environmental stress hypothesis in children with and without developmental coordination disorder. Psychology of Sport and Exercise, 37, 244–250.

[jcpp13001-bib-0032] Lingam, R. , Jongmans, M.J. , Ellis, M. , Hunt, L.P. , Golding, J. , & Emond, A. (2012). Mental health difficulties in children with developmental coordination disorder. Pediatrics, 129, e882–e891. 22451706 10.1542/peds.2011-1556

[jcpp13001-bib-0033] Mancini, V.O. , Rigoli, D. , Cairney, J. , Roberts, L.D. , & Piek, J.P. (2016). The elaborated environmental stress hypothesis as a framework for understanding the association between motor skills and internalizing problems: A mini‐review. Developmental Psychology, 7, 239. 10.3389/fpsyg.2016.00239 PMC4763061 26941690

[jcpp13001-bib-0034] Mancini, V. , Rigoli, D. , Roberts, L. , & Piek, J. (2018). Motor skills and internalizing problems throughout development: An integrative research review and update of the environmental stress hypothesis research. Research in Developmental Disabilities. Advance online publication. 10.1016/j.ridd.2018.07.003. 30054197

[jcpp13001-bib-0035] Martin, N.C. , Piek, J.P. , & Hay, D. (2006). DCD and ADHD: A genetic study of their shared aetiology. Human Movement Science, 25, 110–124. 16442650 10.1016/j.humov.2005.10.006

[jcpp13001-bib-0036] Missiuna, C. (1994). Motor skill acquisition in children with developmental coordination disorder. Adapted Physical Activity Quarterly, 11, 214–235.

[jcpp13001-bib-0037] Missiuna, C. , Cairney, J. , Pollock, N. , Campbell, W. , Russell, D.J. , Macdonald, K. , … & Cousins, M. (2014). Psychological distress in children with developmental coordination disorder and attention‐deficit hyperactivity disorder. Research in Developmental Disabilities, 35, 1198–1207. 24559609 10.1016/j.ridd.2014.01.007

[jcpp13001-bib-0038] Missiuna, C. , & Campbell, W.N. (2014). Psychological aspects of developmental coordination disorder: Can we establish causality? Current Developmental Disorders Reports, 1, 125–131.

[jcpp13001-bib-0039] Missiuna, C. , Moll, S. , King, S. , King, G. , & Law, M. (2007). A trajectory of troubles: Parents’ impressions of the impact of developmental coordination disorder. Physical & Occupational Therapy in Pediatrics, 27, 81–101. 17298942

[jcpp13001-bib-0040] Missiuna, C. , Pollock, N.A. , Levac, D.E. , Campbell, W.N. , Whalen, S.D.S. , Bennett, S.M. , … & Russell, D.J. (2012). Partnering for Change: An innovative school‐based occupational therapy service delivery model for children with developmental coordination disorder. Canadian Journal of Occupational Therapy, 79, 41–50. 10.2182/cjot.2012.79.1.6 22439291

[jcpp13001-bib-0041] Moher, D. , Liberati, A. , Tetzlaff, J. , Altman, D. G. , & The Prisma Group (2009). Preferred reporting items for systematic reviews and meta‐analyses: The PRISMA Statement. PLOS Medicine, 6, e1000097. 19621072 10.1371/journal.pmed.1000097 PMC2707599

[jcpp13001-bib-0042] Norman, G. , Sloan, J. , & Wyrwich, K. (2003). Interpretation of changes in health‐related quality of life: The remarkable universality of half a standard deviation. Medical Care, 41, 582–592. 12719681 10.1097/01.MLR.0000062554.74615.4C

[jcpp13001-bib-0043] Pearsall‐Jones, J.G. , Piek, J.P. , Rigoli, D. , Martin, N.C. , & Levy, F. (2011). Motor disorder and anxious and depressive symptomatology: A monozygotic co‐twin control approach. Research in Developmental Disabilities, 32, 1245–1252. 21349687 10.1016/j.ridd.2011.01.042

[jcpp13001-bib-0044] Piek, J.P. , Bradbury, G.S. , Elsley, S.C. , & Tate, L. (2008). Motor coordination and social–emotional behaviour in preschool‐aged children. International Journal of Disability, Development and Education, 55, 143–151.

[jcpp13001-bib-0045] Piek, J.P. , Rigoli, D. , Pearsall‐Jones, J.G. , Martin, N.C. , Hay, D.A. , Bennett, K.S. , & Levy, F. (2007). Depressive symptomatology in child and adolescent twins with attention‐deficit hyperactivity disorder and/or developmental coordination disorder. Twin Research and Human Genetics, 10, 587–596. 17708700 10.1375/twin.10.4.587

[jcpp13001-bib-0046] Pinquart, M. , & Shen, Y. (2011a). Anxiety in children and adolescents with chronic physical illnesses: A meta‐analysis. Acta Paediatrica, 100, 1069–1076. 21332786 10.1111/j.1651-2227.2011.02223.x

[jcpp13001-bib-0047] Pinquart, M. , & Shen, Y. (2011b). Depressive symptoms in children and adolescents with chronic physical illness: An updated meta‐analysis. Journal of Pediatric Psychology, 36, 375–384. 21088072 10.1093/jpepsy/jsq104

[jcpp13001-bib-0048] Poole, K.L. , Schmidt, L.A. , Missiuna, C. , Saigal, S. , Boyle, M.H. , & Van Lieshout, R.J. (2016). Childhood motor coordination and adult psychopathology in extremely low birth weight survivors. Journal of Affective Disorders, 190, 294–299. 26544612 10.1016/j.jad.2015.10.031

[jcpp13001-bib-0049] Poulsen, A.A. , Ziviani, J.M. , & Cuskelly, M. (2006). General self‐concept and life satisfaction for boys with differing levels of physical coordination: The role of goal orientations and leisure participation. Human Movement Science, 25, 839–860. 16859792 10.1016/j.humov.2006.05.003

[jcpp13001-bib-0050] Poulsen, A.A. , Ziviani, J.M. , Cuskelly, M. , & Smith, R. (2007). Boys with developmental coordination disorder: Loneliness and team sports participation. American Journal of Occupational Therapy, 61, 451–462. 17685178 10.5014/ajot.61.4.451

[jcpp13001-bib-0051] Pratt, M.L. , & Hill, E.L. (2011). Anxiety profiles in children with and without developmental coordination disorder. Research in Developmental Disabilities, 32, 1253–1259. 21377831 10.1016/j.ridd.2011.02.006

[jcpp13001-bib-0052] Rasmussen, P. , & Gillberg, C. (2000). Natural outcome of ADHD with developmental coordination disorder at age 22 years: A controlled, longitudinal, community‐based study. Journal of the American Academy of Child & Adolescent Psychiatry, 39, 1424–1431. 11068898 10.1097/00004583-200011000-00017

[jcpp13001-bib-0053] Raz‐Silbiger, S. , Lifshitz, N. , Katz, N. , Steinhart, S. , Cermak, S.A. , & Weintraub, N. (2015). Relationship between motor skills, participation in leisure activities and quality of life of children with developmental coordination disorder: Temporal aspects. Research in Developmental Disabilities, 38, 171–180. 25589477 10.1016/j.ridd.2014.12.012

[jcpp13001-bib-0054] Rigoli, D. , Kane, R.T. , Mancini, V. , Thornton, A. , Licari, M. , Hands, B. , … & Piek, J. (2017). The relationship between motor proficiency and mental health outcomes in young adults: A test of the environmental stress hypothesis. Human Movement Science, 53, 16–23. 27697306 10.1016/j.humov.2016.09.004

[jcpp13001-bib-0055] Rigoli, D. , Piek, J.P. , & Kane, R. (2012). Motor coordination and psychosocial correlates in a normative adolescent sample. Pediatrics, 129, e892–e900. 22451714 10.1542/peds.2011-1237

[jcpp13001-bib-0056] Rivard, L.M. , Missiuna, C. , Hanna, S. , & Wishart, L. (2011). Understanding teachers’ perceptions of the motor difficulties of children with developmental coordination disorder (DCD). British Journal of Educational Psychology, 77, 633–648. 10.1348/000709906X159879 17908379

[jcpp13001-bib-0057] Rosenthal, R. (1979). The file drawer problem and tolerance for null results. Psychological Bulletin, 86, 638.

[jcpp13001-bib-0058] Sanderson, S. , Tatt, I.D. , & Higgins, J.P. (2007). Tools for assessing quality and susceptibility to bias in observational studies in epidemiology: A systematic review and annotated bibliography. International Journal of Epidemiology, 36, 666–676. 17470488 10.1093/ije/dym018

[jcpp13001-bib-0059] Schoemaker, M.M. , & Kalverboer, A.F. (1994). Social and affective problems of children who are clumsy: How early do they begin? Adapted Physical Activity Quarterly, 11, 130–140.

[jcpp13001-bib-0060] Sigurdsson, E. , van Os, J. , Fombonne, E. , Sigurdsson, E. , Van Os, J. , & Fombonne, E. (2002). Are impaired childhood motor skills a risk factor for adolescent anxiety? Results from the 1958 U.K. birth cohort and the National Child Development Study. American Journal of Psychiatry, 159, 1044–1046. 12042195 10.1176/appi.ajp.159.6.1044

[jcpp13001-bib-0061] Skinner, R.A. , & Piek, J.P. (2001). Psychosocial implications of poor motor coordination in children and adolescents. Human Movement Science, 20, 73–94. 11471399 10.1016/s0167-9457(01)00029-x

[jcpp13001-bib-0062] Stephenson, E.A. , & Chesson, R.A. (2008). ‘Always the guiding hand’: Parents' accounts of the long‐term implications of developmental co‐ordination disorder for their children and families. Child: Care, Health and Development, 34, 335‐343. 10.1111/j.1365-2214.2007.00805.x 18410640

[jcpp13001-bib-0063] Stroup, D.F. , Berlin, J.A. , Morton, S.C. , Olkin, I. , Williamson, G.D. , Rennie, D. , … & Thacker, S.B. (2000). Meta‐analysis of observational studies in epidemiology: A proposal for reporting. Meta‐analysis Of Observational Studies in Epidemiology (MOOSE) group. JAMA, 283, 2008–2012. 10789670 10.1001/jama.283.15.2008

[jcpp13001-bib-0064] Trautmann, S. , Rehm, J. , & Wittchen, H.U. (2016). The economic costs of mental disorders. EMBO Reports, 17, 1245–1249. 27491723 10.15252/embr.201642951 PMC5007565

[jcpp13001-bib-0065] Tseng, M.H. , Howe, T.H. , Chuang, I.C. , & Hsieh, C.L. (2007). Cooccurrence of problems in activity level, attention, psychosocial adjustment, reading and writing in children with developmental coordination disorder. International Journal of Rehabilitation Research, 30, 327–332. 17975453 10.1097/MRR.0b013e3282f144c7

[jcpp13001-bib-0066] Twenge, J.M. , & Nolen‐Hoeksema, S. (2002). Age, gender, race, socioeconomic status, and birth cohort differences on the children's depression inventory: A meta‐analysis. Journal of Abnormal Psychology, 111, 578–588. 12428771 10.1037//0021-843x.111.4.578

[jcpp13001-bib-0067] Voils, C.I. , Crandell, J.L. , Chang, Y. , Leeman, J. , & Sandelowski, M. (2011). Combining adjusted and unadjusted findings in mixed research synthesis. Journal of Evaluation in Clinical Practice, 17, 429–434. 21040243 10.1111/j.1365-2753.2010.01444.x PMC3063329

[jcpp13001-bib-0068] Wagner, M. (2017). Considerations on the assessment of developmental coordination disorder and the elaboration of related contextual pathways. Developmental Medicine & Child Neurology, 59, 891–892. 28650077 10.1111/dmcn.13490

[jcpp13001-bib-0069] Wagner, M. , Bös, K. , Jascenoka, J. , Jekauc, D. , & Petermann, F. (2012). Peer problems mediate the relationship between developmental coordination disorder and behavioral problems in school‐aged children. Research in Developmental Disabilities, 33, 2072–2079. 22750362 10.1016/j.ridd.2012.05.012

[jcpp13001-bib-0070] Wagner, M. , Jekauc, D. , Worth, A. , & Woll, A. (2016). Elaboration of the environmental stress hypothesis‐results from a population‐based 6‐year follow‐up. Frontiers in Psychology, 7, 1904. 28018254 10.3389/fpsyg.2016.01904 PMC5156825

[jcpp13001-bib-0071] Watson, L. , & Knott, F. (2006). Self‐esteem and coping in children with developmental coordination disorder. British Journal of Occupational Therapy, 69, 450–456.

[jcpp13001-bib-0072] Wells, G.A. , Shea, B. , O'Connell, D. , Peterson, J. , Welch, V. , Losos, M. , & Tugwell, P. (2011). The Newcastle‐Ottawa Scale (NOS) for assessing the quality of nonrandomised studies in meta‐analyses. Available from http://www.ohri.ca/programs/clinical_epidemiology/oxford.asp [last accessed 10 May 2018].

[jcpp13001-bib-0073] Wilson, B.N. , Neil, K. , Kamps, P.H. , & Babcock, S. (2013). Awareness and knowledge of developmental co‐ordination disorder among physicians, teachers and parents. Child: Care, Health and Development, 39, 296–300. 10.1111/j.1365-2214.2012.01403.x PMC3579234 22823542

[jcpp13001-bib-0074] Wilson, A. , Piek, J.P. , & Kane, R. (2013). The mediating role of social skills in the relationship between motor ability and internalizing symptoms in pre‐primary children. Infant and Child Development, 22, 151–164.

[jcpp13001-bib-0075] Wilson, P.H. , Ruddock, S. , Smits‐Engelsman, B. , Polatajko, H. , & Blank, R. (2013). Understanding performance deficits in developmental coordination disorder: A meta‐analysis of recent research. Developmental Medicine and Child Neurology, 55, 217–228. 23106668 10.1111/j.1469-8749.2012.04436.x

[jcpp13001-bib-0076] Zwicker, J.G. , Harris, S.R. , & Klassen, A.F. (2013). Quality of life domains affected in children with developmental coordination disorder: A systematic review. Child: Care Health and Development, 39, 562–580. 10.1111/j.1365-2214.2012.01379.x 22515477

