# Supplementary material for: Research Review: Internalising symptoms in developmental coordination disorder: a systematic review and meta‐analysis
Source: J Child Psychol Psychiatry. 2018 Nov 28;60(6):606–21. doi: 10.1111/jcpp.13001 (PMC7379561; doi:10.1111/jcpp.13001)

**Appendix S1.** Summary of search terms used.

The search included terms related to DCD (*developmental coordination disorder, DCD, dyspraxi*, motor skills disorder, coordination difficult*, coordination problem*, clumsy, clumsiness, motor proficiency, motor competence, motor difficult*, motor impairment, motor dysfunction, perceptual motor difficult*, perceptual motor impairment, motor skills disorder, motor learning difficult*, motor learning problem*, motor problem*, movement disorder, psychomotor disorder*)

Combined with terms related to internalising symptoms (*internali*, anxi*, depress*, mood, mental health, mental illness, mental disorder, emotional problem*, psychopatholog**).

**MEDLINE (EBSCO interface) search strategy**

The details of the full search strategy used on the MEDLINE database are provided below:

((TI ( “developmental coordination disorder” OR DCD OR dyspraxi* OR “motor skills disorder” OR “coordination difficult*” OR “coordination problem*” OR clumsy OR clumsiness OR “motor proficiency” OR “motor competence” OR “motor difficult*” OR “motor impairment” OR “motor dysfunction” OR “perceptual motor difficult*” OR “perceptual motor impairment” OR “motor skills disorder” OR “motor learning difficult*” OR “motor learning problem*” OR “motor problem*” OR “movement disorder” OR “psychomotor disorder” )) OR (AB ( “developmental coordination disorder” OR DCD OR dyspraxi* OR “motor skills disorder” OR “coordination difficult*” OR “coordination problem*” OR clumsy OR clumsiness OR “motor proficiency” OR “motor competence” OR “motor difficult*” OR “motor impairment” OR “motor dysfunction” OR “perceptual motor difficult*” OR “perceptual motor impairment” OR “motor skills disorder” OR “motor learning difficult*” OR “motor learning problem*” OR “motor problem*” OR “movement disorder” OR “psychomotor disorder”))) AND ((TI ( internali* OR anxi* OR depress* OR mood OR "mental health" OR "mental illness" OR "mental disorder" OR "emotional problem*" OR psychopatholog*)) OR (AB (internali* OR anxi* OR depress* OR mood OR "mental health" OR "mental illness" OR "mental disorder" OR "emotional problem*" OR psychopatholog* )))

*No limits applied.*

**Figure S1.** Funnel plot of effect sizes and standard error.


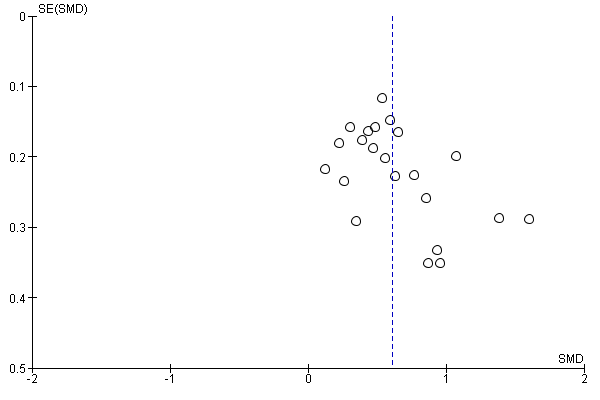

Supplement: Supplementary file 1 — Appendix S1. Summary of search terms used. Figure S1. Funnel plot of effect sizes and standard error. [file JCPP-60-606-s001.docx]
